# Computational Systems Biology Methods in Molecular Biology, Chemistry Biology, Molecular Biomedicine, and Biopharmacy

**DOI:** 10.1155/2014/746814

**Published:** 2014-04-09

**Authors:** Yudong Cai, Julio Vera González, Zengrong Liu, Tao Huang

**Affiliations:** ^1^Institute of Systems Biology, Shanghai University, Shanghai 200444, China; ^2^Department of Systems Biology, University of Rostock, Rostock 18051, Germany; ^3^Department of Mathematics, College of Science, Shanghai University, Shanghai 200444, China; ^4^Department of Genetics and Genomics Sciences, Mount Sinai School of Medicine, New York, NY 10029, USA

In the postgenomic era, the large-scale data, such as genome sequences, mRNA sequences, and protein sequences, increase rapidly. It is desired to develop the computational approaches that can derive and analyze useful information from them to promote the development of biomedicine and drug design. Meanwhile, in order to understand how protein-protein interactions and other complex interactions in a living system get integrated in complex nonlinear networks and regulate cell function, a new discipline, called “Systems Biology”, is created.

In this special issue, 11 interesting studies were included. Several novel computational methods for systems biology were proposed for the first time and some intriguing biological findings were reported in large scale experiments.

J. Cao and L. Xiong studied the protein sequence classification using the single hidden layer feed forward neural network (SLFN). Two algorithms, the basic extreme learning machine (ELM) and the optimal pruned ELM (OP-ELM), were adopted as the learning algorithms for the ensemble based SLFNs. Their methods outperformed back propagation (BP) neural network and support vector machine (SVM).

Y. F. Gao et al. proposed a novel prediction method based on drug and compound ontology information extracted from ChEBI to identify drugs target groups, from which the kind of functions of a drug may be deduced. Their overall prediction accuracy on the training dataset was 83.12%, while it was 87.50% on the test dataset. The study may become an inspiration to solve the problems of this sort and bridge the gap between ChEBI ontology and drugs target groups.

Z. Li et al. developed a computational method to predict retinoblastoma (RB) related genes. RB is the most common primary intraocular malignancy usually occurring in childhood. Their method was based on dagging, with the maximum relevance minimum redundancy (mRMR) method followed by incremental feature selection (IFS). The RB and non-RB genes can be classified with Gene Ontology enrichment scores and KEGG enrichment scores. This method can be generalized to predict the other cancer related genes as well.

Y. Jiang et al. proposed a method to identify gastric cancer genes by first applying the shortest path algorithm to protein-protein interaction network and then filtering the shortest path genes based permutation betweenness. Many identified candidate genes were involved in gastric cancer related biological processes. Their study gives a new insight for studying gastric cancer.

T. Zhang et al. proposed a computational method for gene phenotypes prediction. Their method regarded the multiphenotype as a whole network which can rank the possible phenotypes associated with the query protein and showed more comprehensive view of the protein's biological effects. The performance of their method was better than dagging, random forest, and sequential minimal optimization (SMO).

Q. Zou et al. reviewed the network based disease gene identification methods, such as CIPHER, RWRH, Prince, Meta-path, Katz, Catapult, Diffusion Kernel, and ProDiGe and compared their performance. Some advices about software choosing and parameter setting were provided. They also analyzed the core problems and challenges of these methods and discussed future research direction.

G. S.V. McDowell et al. developed a bioinformatics tool, Visualization and Phospholipid Identification (VaLID), to search and visualize the 1,473,168 phospholipids from the VaLID database. Each phospholipid can be generated in skeletal representation. VaLID is freely available and responds to all users through the CTPNL resources website at http://neurolipidomics.com/resources.html and http://neurolipidomics.ca/.

X. Lai et al. proposed a systems biology approach combining database-oriented network reconstruction, data-driven modeling, and model-driven experiments to study the regulatory role of miRNAs in coordinating gene expression. They illustrate the method by reconstructing, modeling and simulating the miRNA network regulating p21. Their model can be used to study the effect of different miRNA expression profiles and cooperative target regulation on p21 expression levels in different biological contexts and phenotypes.

P. Cui et al. analyzed the genome-wide relationship between chromatin features and chromatin accessibility in DNase I hypersensitive sites. They found that these features show distinct preference to localize in open chromatin. Their study provides new insights into the true biological phenomena and the combinatorial effects of chromatin features on differential DNase I hypersensitivity.

L. Zhu et al. sequenced the transcriptome of* Sophora japonica* Linn (Chinese scholar tree), a shrub species belonging to the subfamily Faboideae of the pea family Fabaceae. Approximately 86.1 million high-quality reads were generated and assembled de novo into 143010 unique transcripts and 57614 unigenes. The transcriptome data of* S. japonica* from this study represents first genome-scale investigation of gene expressions in Faboideae plants.

Y. Wang et al. characterized the microsatellite pattern in the* V. volvacea* genome and compared it with microsatellites found in the genomes of four other edible fungi:* Coprinopsis cinerea, Schizophyllum commune, Agaricus bisporus,* and* Pleurotus ostreatus*. A total of 1346 microsatellites have been identified with mononucleotides, the most frequent motif. Their analysis suggested a possible relationship between the most frequent microsatellite types and the genetic distance between the five fungal genomes.

With the current exponential increase of biological and biomedical high-throughput data generated, in the future we will see how methodologies like the ones described in this special issue become absolutely necessary. But furthermore we need to know how methodologies pertaining data analysis, network reconstruction, and modeling get together (a) to make possible the integration of massive, multiple-type quantitative high-throughput data and (b) to understand how cell phenotypes emerge from large, multilevel and structurally complex biochemical regulatory networks.

*Yudong Cai* *Julio Vera González* *Zengrong Liu* *Tao Huang*

